# Biochemical and inflammatory modifications after switching to dual antiretroviral therapy in HIV-infected patients in Italy: a multicenter retrospective cohort study from 2007 to 2015

**DOI:** 10.1186/s12879-018-3198-2

**Published:** 2018-06-25

**Authors:** Eugenia Quiros-Roldan, Paola Magro, Elena Raffetti, Ilaria Izzo, Alessandro Borghetti, Francesca Lombardi, Annalisa Saracino, Franco Maggiolo, Francesco Castelli, for the MASTER Cohort, F. Castelli, G. Carosi, E. Quiros-Roldan, G. Paraninfo, C. Torti, R. Cauda, S. Di Giambenedetto, M. Fabbiani, M. Colafigli, F. Maggiolo Ospedali Riuniti, A. Scalzini, F. Castelnuovo, I. El Hamad, F. Mazzotta, S. Locaputo, N. Marino, P. Pierotti, M. Di Pietro, C. Blè, F. Vichi, L. Sighinolfi, G. Angarano, N. Ladisa, L. Monno, P. Maggi, A. Pan, S. Costarelli, A. Gori, G. Lapadula, M. Puoti, P. Viale, V. Colangeli, M. Borderi

**Affiliations:** 1 Department of Clinical and Experimental Sciences, University of Brescia, Brescia, Italy; 2 Department of Public Health Sciences, Karolinska Institutet, Stockholm, Sweden; 3 Infectious and Tropical Diseases Unit, Spedali Civili, Brescia, Italy; 4 Institute of Clinical Infectious Diseases, Catholic University of Sacred Heart, Rome, Italy; 5 Clinic of Infectious Diseases, University Hospital Policlinico, Bari, Italy; 6 Division of Infectious Diseases, AO Giovanni XXIII, Bergamo, Italy

**Keywords:** HIV, Switch, Dual-therapy, Inflammation, Antiretroviral therapy

## Abstract

**Background:**

Triple-drug regimens are the gold standard for HIV therapy. Nucleos(t)ide reverse transcriptase inhibitors (NRTIs) reducing regimens are used to decrease drugs toxicity, exposure and costs. Aim of our study was to evaluate trends of biochemical and inflammatory indices in patients switching to dual therapy (DT).

**Methods:**

We included patients that a) switched to a DT from 2007 to 2015 from a tenofovir/abacavir-based triple regimen b) previously maintained a triple and c) subsequently a dual regimen for 12 months with virological suppression. We retrieved data measured at 5 points (at the switch, 6 and 12 months before and after switch). We used platelet-to-lymphocyte ratio (PLR), neutrophil-to-lymphocyte ratio (NLR) and CD4/CD8 ratio as inflammatory indices. We assessed temporal trends of viro-immunological, biochemical and inflammatory parameters.

**Results:**

Overall, 364 and 65 patients switched from a tenofovir- and an abacavir-triple regimen, respectively.

In the tenofovir-reducing group, creatinine clearance and lipids raised after the switch. There was a significant increase in both CD4+ cells and CD4/CD8. CD8+ cells rose after the switch, while opposite trend was found for PLR.

In the abacavir-reducing group total lipids showed a decrease during the first 6 months after the switch and then stabilized. An increase of CD4+ and a decrease of CD8+ cells was observed during the study period, although not statistically significant. While CD4/CD8 remained stable after simplification, PLR decreased significantly after 6 months, then returning to baseline.

CD8+ cells increased in the tenofovir-reducing group despite a viro-immunological response. Intriguingly, PLR decreased, maintaining this trend for 12 and 6 months after tenofovir and abacavir interruption respectively.

**Conclusions:**

Increased PLR has been linked to hypercholesterolemia and metabolic-syndrome, while high CD8+ cells count to increased risk of non-AIDS-related events regardless of CD4 T-cell recovery and to virological failure. Whether these findings may have clinical implications, and which role DT plays on the immune system and on inflammation should be further investigated.

## Background

Since the start of the epidemic in the early eighties, huge efforts have been made in order to develop effective therapies against HIV virus [1]. Triple-drug regimens have proved their efficacy in controlling viral replication [2] and are now considered the gold standard for the treatment of HIV infection both in antiretroviral-naïve and in antiretroviral-experienced patients. These regimens normally include two nucleos(t)ide reverse transcriptase inhibitors (NRTIs) as a backbone, plus one core agent drug from another class: protease inhibitors (PIs), integrase inhibitors (INSTIs) or nucleoside reverse transcriptase inhibitors (NNRTIs) [3–5]. Thanks to these therapies, patients with HIV are now living longer, though experiencing a higher prevalence of aging-related comorbidities, such as metabolic disorders, renal, cardiovascular and liver diseases in addition to neurocognitive impairment [6]. Whether these events are due to adverse effects of long-term use of combined antiretroviral therapy (cART) remains a matter of concern [7]. Tenofovir disoproxil fumarate (TDF) and abacavir (ABC), respectively co-formulated with emtricitabine and lamivudine, have been the two NRTIs combinations most frequently used in clinical practice [5]. Both these NRTIs are well tolerated, although adverse effects often occur in patients who take them chronically. Indeed, TDF has been associated with acute and chronic renal impairment, small molecular weight proteinuria, nephrogenic diabetes insipidus, nephrotic syndrome, Fanconi syndrome [8, 9] and reduction in bone mineral density [10].

Moreover, yet still controversial [11, 12], ABC has been associated with an increased cardiovascular risk [13, 14]. Recently, NRTIs reducing regimens are used as an alternative approach in clinical practice to decrease drugs toxicities, exposure and costs in both cART-naïve and experienced patients [15–17].

Due to the different pharmacokinetic characteristics of each drug, triple drugs regimens offer more chances of an adequate tissue penetrance and distribution, in order to achieve a wider suppression of HIV replication [18]. The efficacy of simplified regimens needs to be addressed by further studies, and so the effects of reducing strategies on systemic inflammation and immunoactivation.

Given that certain dual therapy regimens have already proved to have good virological outcomes when compared to standard triple therapy in cART-experienced patient [19], aim of our study was to evaluate the trends of simplification to dual therapy in a cohort of patients coming from TDF or ABC-containing triple regimens, focusing on biochemical and inflammatory changes.

## Methods

The Italian MASTER cohort is a hospital-based multicenter, open HIV cohort established in the mid-1990s, with retrospective patients enrollment from 1986 to 1997 and prospective recruitment subsequently. Patients are recruited from 8 HIV clinics in Italy. Inclusion criteria include a positive HIV-1 or HIV-2 antibody test, or a positive HIVRNA, and being in care in one of the participating centers. As long as the cohort first objective was to represent the epidemiological and clinical trend of HIV infection through the country, enrollment in MASTER is independent from the HIV disease stage, degree of immunosuppression or use of antiretroviral therapy. Clinical data are recorded for each patient in an electronic database every three/four months and a data check is performed at a central level every six months [20]. In the present study, we included all patients that a) switched to a dual therapy between January 2007 and June 2015 coming from a triple regimen that included tenofovir/emtricitabine (TDF/FTC) or abacavir/lamivudine (ABC/3TC) b) had been durably on a ABC/TDF-containing triple therapy for 12 months before switching and c) have maintained dual therapy for 12 months.

In the switch to dual therapy, those who were prescribed regimen 1 to 3 (Table 1) suspended TDF and ABC respectively co-formulated with emtricitabine and lamivudine. In these patients the PI was maintained and associated with 3TC. Those who were switched to regimen 4 suspended both the NRTIs, starting one INSTI and maintaining the PI.

**Table 1:** Demographic and clinical features at switch to dual therapy

Variables	From tenofovir/emtricitabine n (%) (*n* = 364)	From abacavir/lamivudine n (%) (*n* = 65)	*p*-value^a^
Male	249 (68.4)	52 (80.0)	0.060
Age, in years, mean (SD)	48.1 (10.2)	51.0 (10.4)	
< 40	65 (17.9)	5 (7.7)	0.093
40–49	145 (39.8)	26 (40.0)	
≥ 50	154 (42.3)	34 (52.3)	
Intravenous Drug Use	85 (27.6)	15 (25.9)	0.953
HCV co-infection	134 (36.8)	24 (36.9)	0.987
CD4 cell count, cell/mm3, mean (SD)	631.6 (295.6)	678.1 (342.2)	
< 200	15 (4.9)	1 (1.8)	0.674
200–349	35 (11.4)	8 (14.6)	
350–499	51 (16.7)	8 (14.6)	
≥ 500	205 (67.0)	38 (69.1)	
Positive HIV-RNA, > 37 copies/mL	37 (12.3)	6 (11.3)	0.835
Time (months) in tenofovir/abacavir	110.0 (68.3)	110.4 (78.7)	0.970
Dual regimens:			
Atazanavir/r + 3TC (regimen 1)	121 (33.2)	17 (26.2)	< 0.001
Darunavir/r + 3TC (regimen 2)	84 (23.1)	14 (21.5)	
Lopinavir/r + 3TC (regimen 3)	31 (8.5)	1 (1.5)	
Raltegravir/dolutegravir+PI/r (regimen 4)	128 (35.2)	33 (50.8)	
Number of patients with at least one previous virological failure	79 (21.7)	25 (38.5)	0.004

^a^We used Student t-test for comparison of means Chi-squared test for comparition of proportions

We retrieved data on gender, age, date of enrollment, country of origin, HIV exposure risk and viral hepatitis C co-infection from the MASTER electronic database at the switchover date. The following parameters, measured at 5 different points of follow-up (12 months before, 6 months before, at the switch to dual therapy, 6 months after and 12 months after the switch), were also collected: HIV-RNA, CD4 cell count, CD8 cell count, neutrophils, lymphocytes and platelets counts, total cholesterol, high density lipoprotein (HDL) cholesterol, low density lipoprotein (LDL) cholesterol, triglycerides, serum creatinine and transaminases.

The study was conducted in accordance with the guidelines of the Declaration of Helsinki and the principles of Good Clinical Practice. The study protocol was approved by the local ethics committees on the 4th of August, 2009, reference number 708. Written informed consent was obtained by all patients enrolled [20].

### Outcomes

We evaluated temporal trends of virological and biochemical parameters and inflammatory indices, before and after the switch to dual therapy.

The creatinine clearance (CCr) was estimated from the serum creatinine, weight and height according to the Cockcroft Gault equation [21]. We used platelet-to-lymphocyte ratio (PLR), neutrophil-to-lymphocyte ratio (NLR) and CD4/CD8 ratio as inflammatory indices.

### Statistical analysis

We performed a retrospective, multicenter, case cross-over study [22]. The study period for each subject went from 12 months before to 12 months after the switchover date. The parameters were expressed in means (standard deviations, SDs) or proportions as appropriate, according to the follow-up points (12 months before, 6 months before, at the switch to dual therapy, 6 months after and 12 months after the switch). We compared the characteristics at the moment of switchover between subjects coming from tenofovir-based regimens and those coming from abacavir-based regimens using common statistical tests for comparison of means (Student’s t-test) or proportions (Chi-squared test). We assessed temporal trends of HIV-RNA, CD4 cell count, CD8 cell count total, ALT, AST, cholesterol, HDL, LDL triglycerides, CCr, lymphocytes, neutrophils, platelets, PLR and NLR before and after the switch using mixed models for repeated measures. In the analysis we performed random effects mixed models in order to evaluate the role of time (in months) on the changes in biochemical parameters. These models had a random effect on subjects. Age, gender, previous AIDS event, HCV and HBV co-infection are not potential confounders because they are fixed over time. A sensitivity analysis by multiple imputation for missing data was performed. This method requires that the data are missing at random–not related to the missing values. If this assumption holds, resulting estimates (i.e., regression coefficients and standard errors) will be unbiased with no loss of power. We also stratified the analysis according to the regimens after switching (atazanavir, darunavir, lopinavir-ritonavir and integrase inhibitor regimens). The results of mixed models were expressed in terms of coefficients and their 95% confidence intervals (CIs). The coefficient should be interpreted as in a linear regression model: an increase in one unit in time leads to an increase in one unit in the outcome (biological parameters). We have used month as time unit.

All statistical tests were two-sided, where a level of significance of 0.05 has been assumed. Statistical tests were performed using Stata software version 12.0 (StataCorp, College Station, TX, USA).

## Results

A total of 6067 subjects were followed in the Master Cohort between 2007 and 2015. TDF- and ABC- containing triple regimens were prescribed at least once to 1702 and 745 patients, respectively. Overall, simplification rate to dual therapy per 100 person-years was 8.83 (CI 95% 8.02–9.69) from both TDF- and ABC-containing triple regimens, while it was 7.50 (6.76–8.30) and 1.32 (1.02–1.68) for TDF- and ABC-containing triple regimens, respectively.

A total of 429 subjects (70.2% males) with a mean age of 49.8 years were included in this analysis. Demographic and clinical features are showed in Table 1. Overall, 364 patients switched from a TDF-containing triple regimen and 65 from an ABC-containing triple regimen to a dual therapy.

Patients that discontinued TDF, compared to those suspending ABC, had a lower prevalence of males (68.4% vs 80.0%), subjects with age ≥ 40 years (82.1% vs 92.3%) and experienced less virological failure (HIVRNA > 50 copies/ml) (21.7% vs 38.5%) before the simplification.

Dual regimens prescribed were categorized as follows: atazanavir/ritonavir (RTV) plus 3TC (regimen 1), darunavir/RTV plus 3TC (regimen 2), lopinavir-RTV plus 3TC (regimen 3), and raltegravir or dolutegravir plus ritonavir boosted protease inhibitor (regimen 4).

In the TDF reducing-group, when compared to the ABC reducing-group, there was a higher proportion of subjects that underwent regimen 1 (33.2% vs 26.2%) and regimen 3 (8.5% vs 1.5%), and a lower proportion of subjects in regimen 4 (35.2% vs 50.8%). The proportions of subjects in regimen 2 were similar in the two groups.

### Biological parameters before and after switch in the TDF-reducing therapy group. Table 2

**Fig. 1:**
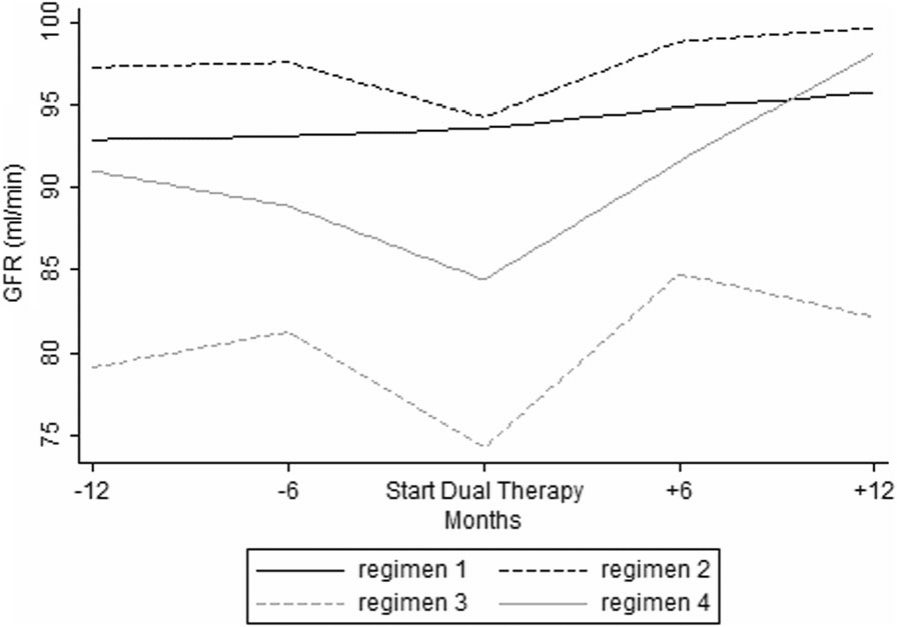
Temporal trends of glomerular filtration rate (CCr) before and after switch in the TDF-reducing therapy group

The CCr significantly decreased from 92.1 to 88.6 ml/min before the switch, and rose from 88.6 to 95.8 ml/min after the switch (*p* < 0.001 for both comparisons). CCr showed a significant biphasic trend with a decrease before the switch and an increase after the switch in all the regimens excluding regimen 1, where CCr was stable around 95 ml/min (*p* = 0.132). The best CCr recovery was observed after switching to the INSTI plus PI regimen (regimen 4) (*p* < 0.001) (Fig. 1).

**Table 2:** Parameters during the follow-up in the TDF-reducing therapy group (*n* = 364). Dual therapy (DT) Alanine transaminase (ALT) Aspartate transaminase (AST) High-density lipoprotein (HDL) Low-density lipoprotein (HDL) Creatinine clearance (CCr) platelet-to-lymphocyte ratio (PLR) neutrophil-to-lymphocyte ratio (NLR)

Parameters	12 months before Mean (SD)	6 months before Mean (SD)	Swicht to DT Mean (SD)	6 months after switch Mean (SD)	12 months after switch Mean (SD)	From 12 months before to switch^a^ Coef. (CI 95%)	p-value	From switch to 12 months after^a^ Coef. (CI 95%)	*p*-value
CD4 cell count, cell/mm3	611.1 (302.8)	630.5 (300.5)	631.6 (295.6)	659.3 (295.9)	699.2 (296.3)	1.9 (0.3,3.5)	0.023	6.8 (4.9,8.8)	< 0.001
CD8 cell count, cell/mm3	900.1 (423.6)	910.2 (439.7)	872.3 (438.6)	928.4 (475.8)	919.2 (474.2)	−1.8 (− 4.9,1.4)	0.272	4.2 (1.2, 7.6)	0.007
ALT (U/l)	40.8 (46.8)	38.4 (33.9)	37.2 (30.4)	38.4 (44.6)	36.6 (35.6)	−0.3 (− 0.7,0.0)	0.045	− 0.0 (− 0.1,0.4)	0.890
AST (U/l)	33.6 (37.4)	30.3 (22.8)	30.3 (24.3)	29 (29.4)	30.2 (26.6)	−0.3 (− 0.6–0.0)	0.028	−0.0 (− 0.3,0.2)	0.760
Total Cholesterol (mg/dl)	184.9 (40.6)	186.9 (40.6)	186 (41.4)	208.4 (45.9)	199.7 (45.5)	0.1 (−0.2,0.4)	0.601	1.5 (1.1,1.9)	< 0.001
HDL Cholesterol (mg/dl)	44.7 (14.8)	45.9 (16.4)	46 (15.9)	49.6 (15.4)	48.9 (15.9)	0.2 (0.0,0.3)	0.009	0.3 (0.2,0.5)	< 0.001
LDL Cholesterol (mg/dl)	111.9 (35)	113.1 (34.5)	114.3 (34)	125.1 (37.2)	120 (38.2)	−0.1 (−0.2, 0.5)	0.534	0.7 (0.2, 1.1)	0.005
Triglycerides (mg/dl)	157.1 (90.9)	160.5 (85.1)	155.7 (105.2)	174.3 (125.3)	176.3 (141.2)	−0.2 (−1.2,0.7)	0.629	1.3 (0.3,2.3)	0.009
CCr (ml/min)	92.1 (25.2)	91.1 (28.5)	88.6 (30.2)	93.2 (28.6)	95.8 (29.7)	−0.4 (−0.5, − 0.2)	< 0.001	0.5 (0.3,0.6)	< 0.001
Lymphocyte, 10^3^/μL	2087 (789)	2100 (767)	2062 (791)	2202 (868)	2236 (867)	−3.3 (−8.0, 3.3)	0.259	16.9 (11.2,22.6)	< 0.001
Neutrophils, 10^3^/μL	4230 (5825)	3435 (1386)	3422 (1344)	3444 (1463)	3560 (1610)	−65.4 (− 127.4–3.4)	0.039	20.3 (− 1.8, 42.4)	0.072
Platelets, 10^3^/μL	216,359 (76022)	216,525 (73144)	218,494 (72903)	214,413 (69168)	214,286 (69895)	−10.9 (− 394.4, 372.96)	0.955	− 420.4 (− 810.2, − 30.5)	0.035
NLR	1.9 (0.9)	1.9 (0.9)	2 (1.1)	1.9 (1.2)	1.9 (0.9)	0.1 (0.0,0.2)	0.067	0.0 (−0.0, 0.0)	0.355
PLR	114.2 (51.5)	113.4 (51.6)	118.7 (59.5)	109.4 (52.8)	105.0 (46.0)	0.3 (−0.1, 0.8)	0.110	−1.2 (−1.6,-0.7)	< 0.001
CD4/CD8	0.79 (0.48)	0.8 (0.5)	0.84 (0.47)	0.81 (0.42)	0.88 (0.43)	0.004 (0.001,0.008)	0.008	0.003 (0.001,0.005)	0.002

In the analysis we performed random effects mixed models in order to evaluate the role of time (in months) on the changes in biochemical parameters

^a^Results remained unchanged when sensitivity analysis by multiple imputation for missing data was performed

**Fig. 2:**
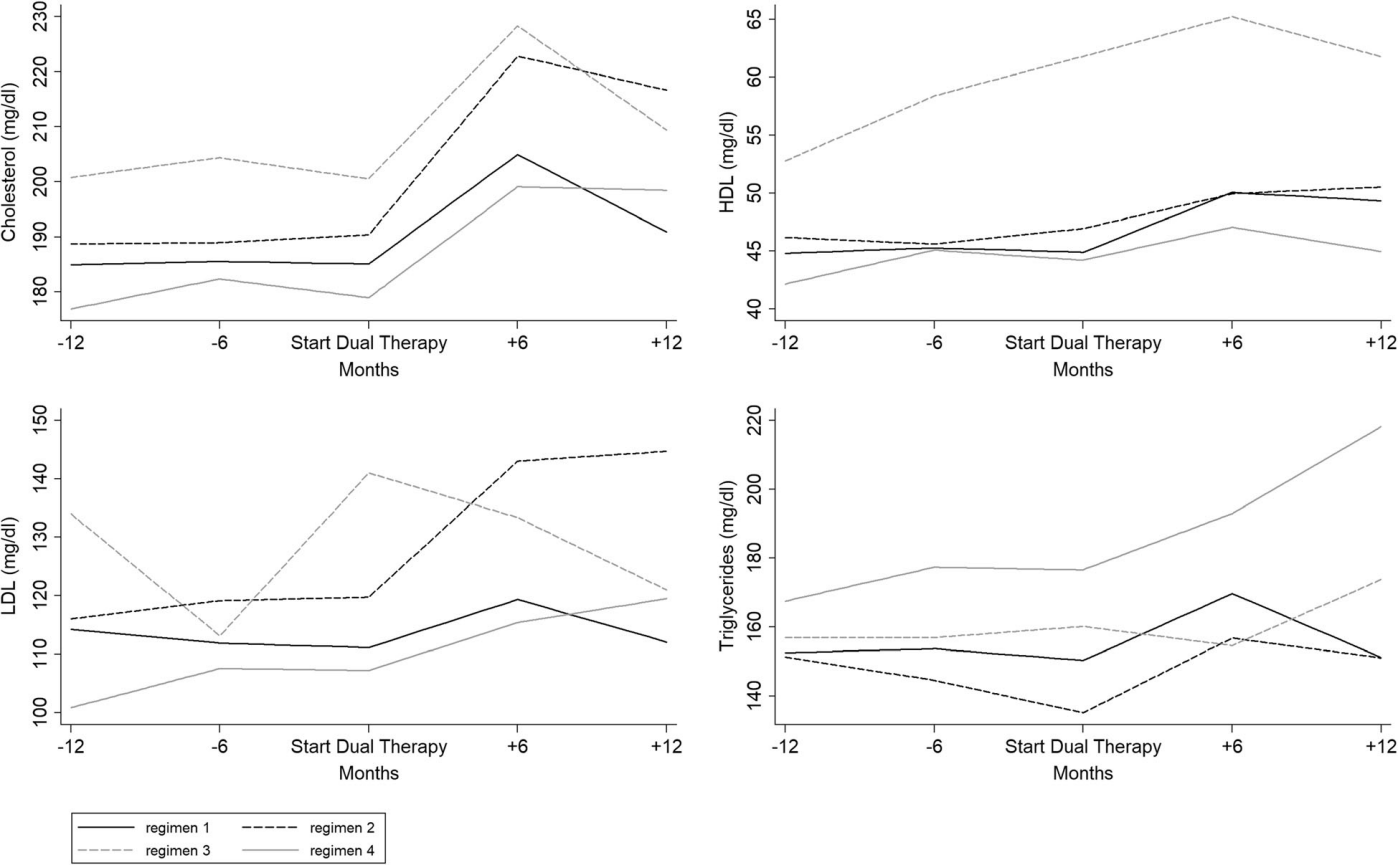
Temporal trends of lipids before and after switch in the TDF-reducing therapy group

With regard to the lipid pattern, total, HDL and LDL cholesterol and triglycerides showed a fluctuation over time with an overall increase mainly during the first 6 months after the switchover to a dual therapy. After switching to dual therapy, the total cholesterol rose significantly in subjects undergoing regimens 1, 2 and 4 (*p* < 0.005 for all comparisons). HDL increased in regimen 1 (*p* < 0.001), whereas LDL rose significantly in regimens 2 and 4 (*p* < 0.001 for both). Triglycerides declined in the year before the switch in regimen 2 from 151.3 to 135.2 mg/ml (*p* = 0.045) and increased after the switch in regimen 4 from 176.7 to 218.2 mg/ml (*p* = 0.022) (Fig. 2).

**Fig. 3:**
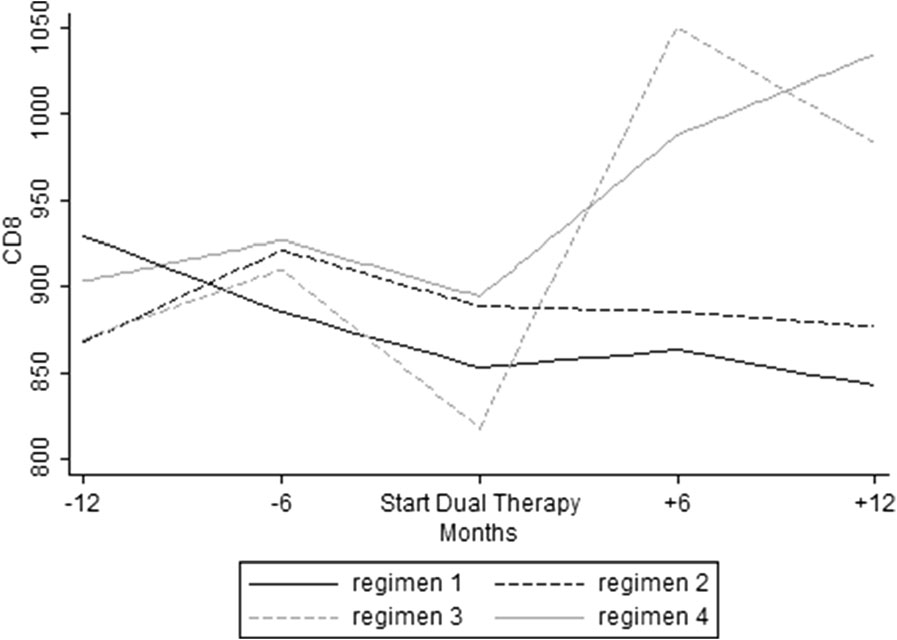
Temporal trends of CD8+ cells before and after switch in the TDF-reducing therapy group

There was a significant increase in both CD4+ cell count and CD4/CD8 over the study period. On the contrary, the CD8+ cell count rose only after the switch from 872.3 to 919.2 cell mm3 (*p* = 0.007). When analyzing the different dual regimens, CD8+ cells trend showed a significant increase only for regimen 4 (*p* = 0.049) after the switch (Fig. 3).

**Fig. 4:**
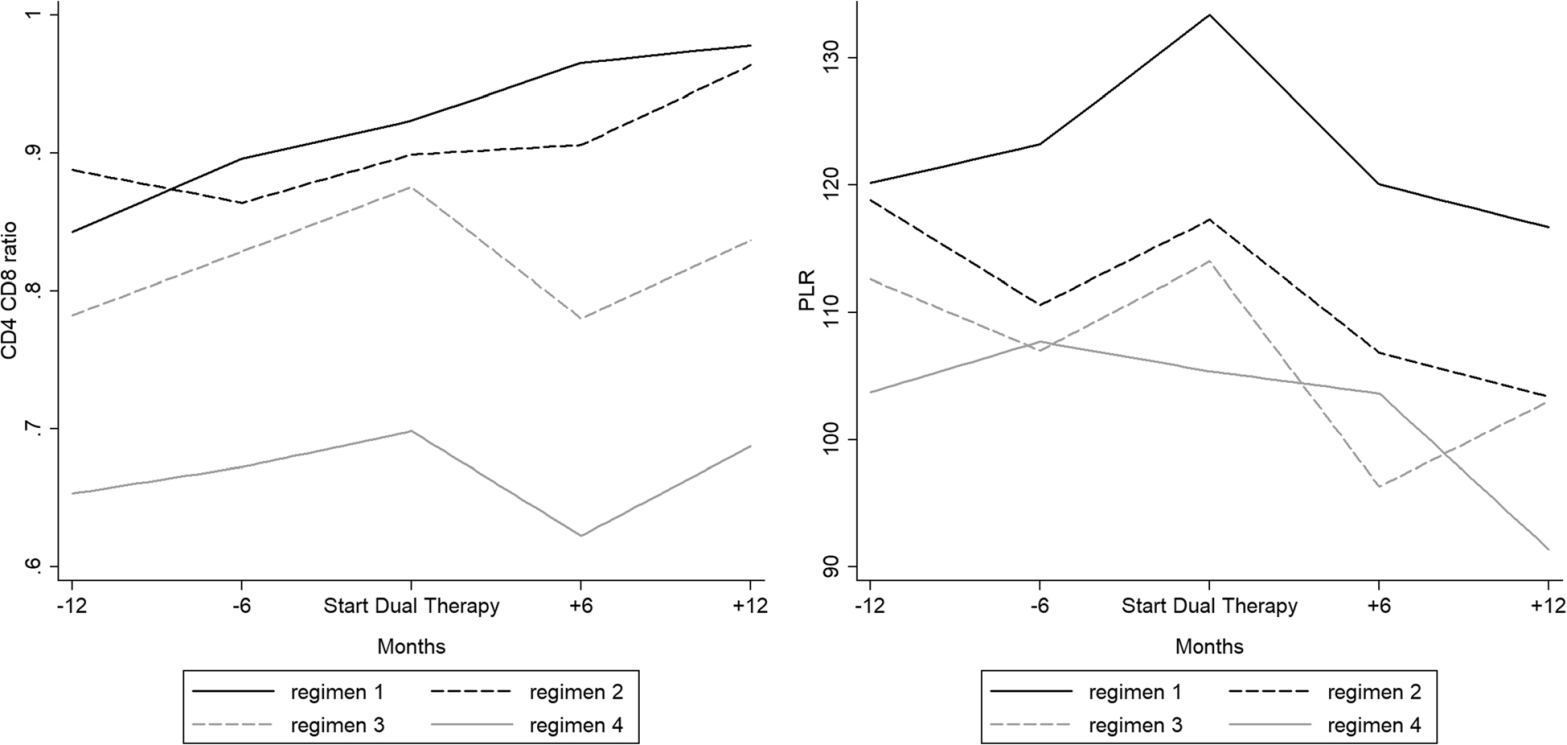
Temporal trends of CD4/CD8 ratio and platelet/lymphocyte ratio (PLR) before and after switch in the TDF-reducing therapy group

Concerning CD4/CD8 ratio (Fig. 4), we observed a persistent rise before and after simplification only for regimens 1 and 2 (*p* < 0.001 for both).

We did not observe any variations of the NLR during the study period. PLR remained stable before and decreased after the switch to dual therapy (from 118.7 to 105.0, *p* < 0.001). When the 4 regimens were analyzed separately, there was a significant reduction of PLR after the switch for regimen 1 (from 133.4 to 116.7, *p* < 0.001), regimen 2 (117.3 to 103.4, *p* = 0.007) and regimen 4 (from 105.4 to 91.4, *p* = 0.038) (Fig. 4).

### Biological parameters before and after switch in the ABC-reducing group. Table 3

Total and LDL cholesterol and triglycerides showed a decrease during the first 6 months after the switch and then stabilized. Anyway, only the changes on total cholesterol were statistically significant. Overall there was an increase of the CD4+ cells and a decrease of CD8+ cells during the study period, although not statistically significant. Moreover, CD4/CD8 remained stable after simplification. There were no significant changes of NLR and PLR over the observational period. Intriguingly, PLR decreased significantly after 6 months from simplification (coeff. -2.07 95% CI -3.43, − 0.71; *p* = 0.003), returning then to baseline levels. Due to the low number of subjects in this group, we didn’t perform a stratified analysis for each regimen.

**Table 3:** Parameters during the follow-up in the ABC-reducing therapy group (*n* = 65). Dual therapy (DT) Alanine transaminase (ALT) Aspartate transaminase (AST) High-density lipoprotein (HDL) Low-density lipoprotein (HDL) Creatinine clearance (CCr) platelet-to-lymphocyte ratio (PLR) neutrophil-to-lymphocyte ratio (NLR)

Parameters	12 months before Mean (SD)	6 months before Mean (SD)	Swicht to DT Mean (SD)	6 months after switch Mean (SD)	12 months after switch Mean (SD)	From 12 months before to switch^a^ Coef. (CI 95%)	*p*-value	From switch to 12 months after^a^ Coef. (CI 95%)	*p*-value
CD4 cell count, cell/mm3	629.9 (327.7)	656.6 (328)	678.1 (342.2)	677.4 (309.6)	658.9 (265.4)	1.3 (−2.3,4.9)	0.481	1.6 (−2.4,5.5)	0.435
CD8 cell count, cell/mm3	1023.5 (473.3)	1069.7 (664)	982.6 (461)	992.6 (526.1)	970.7 (446.6)	−2.5 (− 10.5,5.5)	0.541	− 1.3 (− 7.3,4.6)	0.658
ALT (U/l)	40.1 (41.6)	43.5 (64.2)	44.5 (47.7)	45.9 (55.9)	36.7 (34.8)	0.4 (−0.9,1.6)	0.580	−0.2 (− 1.0,0.5)	0.536
AST (U/l)	30.2 (28.8)	31.2 (42)	29.6 (30)	32.7 (32.4)	30.1 (21.4)	0.1 (−0.8,0.9)	0.860	−0.1 (− 0.6,0.4)	0.761
Total Cholesterol (mg/dl)	218.5 (44.3)	220.8 (51.1)	227.2 (48.8)	218.4 (54.6)	215.5 (39.2)	0.6 (−0.3,1.5)	0.218	−1.6 (−2.6–0.6)	0.003
HDL Cholesterol (mg/dl)	47.2 (17.4)	53.5 (19.6)	49.8 (14.1)	53.9 (19.5)	52.8 (19.7)	0.1 (−0.2,0.4)	0.604	0.2 (−0.1,0.4)	0.168
LDL Cholesterol (mg/dl)	141.1 (38.3)	133.4 (35.5)	145.6 (42.4)	138.4 (30.9)	139.1 (27)	0.2 (−0.9,1.3)	0.742	−0.7 (1.7,0.2)	0.132
Triglycerides (mg/dl)	163.8 (92.2)	180.8 (110.9)	202.8 (115.5)	194.2 (234.4)	172.5 (161.1)	2.4 (0.3,4.6)	0.027	−3.8 (−8.4,0.9)	0.112
CCr (ml/min)	94.5 (31.5)	97 (35.8)	92.2 (26.8)	91.6 (28.5)	92.3 (30.9)	0.0 (−0.4,0.3)	0.852	−0.4 (− 0.8–0.1)	0.041
Lymphocyte, 10^3^ /μL	2201 (662)	2197 (838)	2191 (663)	2256 (686)	2219 (655)	0.2 (−13.7,14.2)	0.972	6.5 (− 7.8,20.8)	0.373
Neutrophils, 10^3^ /μL	3227 (1354)	3462 (1643)	3851 (3336)	3528 (1497)	6059 (10610)	40.7 (−32.7114.0)	0.277	153.3 (−82.1388.7)	0.202
Platelets, 10^3^ /μL	216,173 (63378)	213,125 (68580)	211,927 (59231)	198,250 (60544)	217,611 (59716)	−665.6 (− 1486.0, 154.7)	0.112	98.2 (− 781.3977.8)	0.827
NLR	1.5 (0.6)	1.7 (0.9)	1.8 (1.3)	1.7 (0.6)	1.7 (0.7)	0.2 (−0.2,0.5)	0.367	0.0 (−0.02,0.03)	0.840
PLR	106.9 (46.1)	108.5 (47.7)	104 (36.6)	92.5 (31.2)	107.5 (42.4)	−0.4 (−1.3,0.5)	0.378	−0.2 (− 1.1,0.7)	0.645
CD4/CD8	0.73 (0.42)	0.76 (0.43)	0.82 (0.47)	0.81 (0.42)	0.79 (0.38)	0.005 (0.002,0.008)	0.003	0.002 (−0.001,0.006)	0.155

In the analysis we performed random effects mixed models in order to evaluate the role of time (in months) on the changes in biochemical parameters

^a^Results remained unchanged when sensitivity analysis by multiple imputation for missing data was performed

## Discussion

An improvement in renal function and a worsening in the lipids profile were observed when TDF was removed from triple regimens, while ABC suspension was followed by a significant decrease in total cholesterol only. With regard to the immune reconstitution and to the inflammatory status, CD4+ cell counts and CD4/CD8 raised after simplification, especially in the TDF-reducing group. Intriguingly, CD8+ cells also increased, but only in the TDF-reducing group.

PLR, but not NLR level, decreased after NRTIs reduction. This trend was maintained for 12 months after TDF interruption and for only 6 months after the suspension of ABC.

During the study period, an overall simplification rate to dual therapy of 8.83/100 person-years has been observed. Simplification rate was higher from TDF- than from ABC-containing triple regimens (7.50/100 vs 1.32/100 person-year). Despite TDF-containing triple regimens were the preferred choice for most patients, physicians had suspended TDF more than ABC, as previously described in other studies focused on dual therapies [16]. This could reflect somehow the tendency to switch to a TDF-free dual-therapy regimen because of kidney or bone adverse effects, especially in an aging population. In the near future, this trend could be reverted thanks to the introduction of tenofovir alafenamide (TAF), which is thought to be safer in terms of renal and bone toxic effects [23].

Nowadays, the strategy of reducing the number of drugs in the cART regimen in order to reduce, or prevent, NRTIs-related toxicities and drug-to-drug interactions is not uncommon. Anyway, very few dual regimens are recommended by national and international guidelines [3–5]. Recently, in an Italian geriatrics cohort, it has been pointed out that around 25% of patients received unconventional dual regimens (mainly NRTIs-sparing), reaching an overall viral suppression in about 95% of patients [24]. NRTIs-sparing dual therapies were also the most frequent regimen used in our study (protease inhibitor plus integrase inhibitor). Although this regimen is not mentioned in the guidelines, enough information, from both trials [15, 16] and observational studies [25], have proved their efficacy and safety profile.

As previously observed in other studies [26–28], an improvement of kidney function and a disappearance of the lipid-lowering effect of TDF were shown after TDF discontinuation, while on the other hand, we observed an improvement of lipids profile after ABC suspension. To the best of our knowledge, no previous studies, apart from Di Giambenedetto and Fabbiani et al. [29, 30], specifically focused on the changes in renal function and lipids concentration during the switch from a triple to a dual cART regimen. Anyway, none of these two studies analyzed results from a TDF- or an ABC-stopping therapy separately. In the cohort from Di Giambenedetto, 82% of patients interrupted TDF, and the modifications observed in lipids and in the renal function were similar to ours.

Information on the impact of the various cART regimens on inflammation/immunoactivation remain very limited [31–35]. Actually, there is no consistent evidence about any differences in the reduction of inflammation and immune activation between protease and integrase inhibitors [33, 36, 37]. TAF and TDF (when included in a triple regimen) seem to have an equivalent impact on monocyte activation and on the reduction of systemic inflammation [38]. Moreover, no studies focused on the effects that NRTIs-reducing strategies may have on inflammatory markers in an overall virologically suppressed cART-experienced population.

Here we show that CD8+ cells increased in the group of patients stopping TDF, but not in those stopping ABC. The meaning of this trend in patients switching to a TDF-reducing therapy needs further studies and a longer follow-up in order to discover whether this finding could have clinical and/or virological implications.

Persistent inflammation is a predictor for clinical events and mortality in HIV-infected patients. Anyway, no agreement has been achieved about which one should be the best marker of inflammation in these cases [39, 40]. Persistently elevated CD8+ cells counts during long-term cART have been linked with increased risk of non-AIDS-related events in HIV-infected patients, regardless of CD4 T-cells recovery, and have been related with a chronic inflammation status, and with increased immuneactivation markers such as IL-6, soluble CD14 and d-dimer [39, 41, 42]. Higher CD8+ cells counts at cART initiation, or an increase in CD8+ cell count during cART, seem to be predictive of virological treatment failure [43–45]. In a study from Mussini et al. [46], a lower increase in CD4/CD8 ratio due to a raise in CD8+ cells was found in those switching to a mono/dual-therapy, when compared to those remaining on a standard triple regimen. Anyway, mono and dual therapies were not separated in this analysis, and the comparative group was composed by patients who remained on a triple regimen.

The persistence of HIV replication in sanctuary sites, despite undetectable viremia in plasma, could explain the persistence of systemic inflammation and immune activation, especially because cART penetration into viral reservoirs and sanctuary sites differs among different antiretroviral drugs. NRTIs-reducing strategies could alter the intracellular total cART concentrations when compared to plasma concentrations [20, 47, 48]. Very few data are available about what happens in HIV sanctuaries and reservoirs when simplified cART regimens are used in persistently HIV suppressed patients. ATLAS study showed that a simplification strategy had the same impact on the cellular viral reservoir when compared to triple regimen, but no data are available about immuno-inflammatory trends [49].

Recently, two biomarkers derived from common blood parameters, the neutrophil to lymphocyte ratio (NLR) and the platelet to lymphocyte ratio (PLR), have shown to be indicative of systemic inflammation [50, 51]. In previous studies from the MASTER cohort, we explored the role of NLR and PLR scores as predictors of cardiovascular events, prognosis of cancer and all-cause mortality, proving that they can have a role as positive predictors of these events [52–56].

In the present study, NLR and PLR levels were low throughout the follow-up period. Anyway, we observed a further significant decrease of PLR, but not of NLR levels, after NRTIs interruption. This trend was maintained for 12 months after TDF interruption, and for only 6 months after the discontinuation of ABC. More studies are needed to explore whether these fluctuations may have a clinical significance, or they are a casual feature. Data coming from either HIV-infected and -uninfected persons could explain this variation.

Previously, poor renal function [57] and low levels of vitamin D [58] have been associated with systemic inflammation during HIV infection. In HIV-uninfected people PLR was found to be an independent predictor of 25(OH)D levels along with PTH, calcium, sex and creatinine [59]. In our study, Vitamin D values were not available. Nevertheless, the impact of TDF on bone metabolism and loss of bone mass is known [60]. Whether the decrease of PLR is related with an improvement on the bone turnover and/or of the bone mass as a consequence of TDF interruption, must be deepened.

PLR was also found to be superior to NLR in predicting higher inflammation in end-stage renal disease in HIV-negative patients [61].

The PLR variation following ABC-reducing therapy could be mediated by the lipid profile improvement. Hypercholesterolemia has been linked to systemic and vascular inflammation through several cytokines [62, 63]. Increased PLR has been significantly associated with both presence and severity of metabolic syndrome in HIV-uninfected people [64]. Here, we observed a reduction on total cholesterol during the first 6 months after ABC suspension (maintained also after 12 months) and a concomitant significant decrease of PLR after 6 months, but not after 12 months from ABC suspension. More studies are needed in order to correlate the variations in the lipid profile with this simple and cheap indirect indicator of inflammation.

Obviously, several limitations are present in our study. They include the weakness of a retrospective study. Second, the sample size in the ABC-stopping group and in the several subgroups of dual therapies were relatively small and the results need to be confirmed in larger populations of patients. A further limitation is that associations between PLR and other inflammatory biomarkers were not measured and possible confounders of inflammation were not assessed.

This was not a prospective controlled study, so we cannot draw cause-and-effect relationships from our findings about inflammation. Despite the difficulties in using retrospective study results, strength of our study is the large sample size of a multicenter cohort.

## Conclusions

To conclude, in our study we confirm the disappearance of the lipid-lowering effect and the positive impact on creatinine clearance in those discontinuing TDF, and we observed a decrease of total cholesterol after ABC suspension. Moreover, we found an increase in the CD8+ T-cells besides a PLR reduction in those patients who discontinued TDF and switched to a dual therapy.

Larger controlled studies are required to confirm our findings and to better elucidate the relationship between stopping NRTIs and PLR or- CD8 cells fluctuations. Moreover, a longer follow-up could elucidate whether these findings could correlate with specific clinical events.

## Abbreviations

ALT: Alanine transaminase
AST: Aspartate transaminase
CART: Combined anti-retroviral therapy (cART).
CCr: Creatinine clearance
DT: Dual therapy
HDL: High-density lipoprotein (HDL)
HDL: Low-density lipoprotein
INSTIs: Integrase inhibitors
NLR: neutrophil-to-lymphocyte ratio
NNRTIs: Nucleoside reverse transcriptase inhibitors
NRTIs: Nucleos(t)ide reverse transcriptase inhibitors as a backbone, plus one core agent drug from another class
PI: Protease inhibitors
PLR: Platelet-to-lymphocyte ratio
